# BCAT1 is a New MR Imaging-related Biomarker for Prognosis Prediction in IDH1-wildtype Glioblastoma Patients

**DOI:** 10.1038/s41598-017-17062-1

**Published:** 2017-12-18

**Authors:** Hye Rim Cho, Hyejin Jeon, Chul-Kee Park, Sung-Hye Park, Koung Mi Kang, Seung Hong Choi

**Affiliations:** 1 Department of Radiology, Seoul National University Hospital, Seoul, Korea; 2 Center for Nanoparticle Research, Institute for Basic Science (IBS), Seoul, Korea; 3 Department of Neurosurgery, Seoul National University Hospital, Seoul, Korea; 4 Department of Pathology, Seoul National University Hospital, Seoul, Korea

## Abstract

Isocitrate dehydrogenase 1 (IDH1)-wildtype glioblastoma (GBM) has found to be accompanied with increased expression of branched-chain amino acid trasaminase1 (BCAT1), which is associated with tumor growth and disease progression. In this retrospective study, quantitative RT-PCR, immunohistochemistry, and western blot were performed with GBM patient tissues to evaluate the BCAT1 level. Quantitative MR imaging parameters were evaluated from DSC perfusion imaging, DWI, contrast-enhanced T1WI and FLAIR imaging using a 3T MR scanner. The level of BCAT1 was significantly higher in IDH1-wildtype patients than in IDH1-mutant patients obtained in immunohistochemistry and western blot. The BCAT1 level was significantly correlated with the mean and 95^th^ percentile-normalized CBV as well as the mean ADC based on FLAIR images. In addition, the 95^th^ percentile-normalized CBV from CE T1WI also had a significant correlation with the BCAT1 level. Moreover, the median PFS in patients with BCAT1 expression <100 was longer than in those with BCAT1 expression ≥100. Taken together, we found that a high BCAT1 level is correlated with high CBV and a low ADC value as well as the poor prognosis of BCAT1 expression is related to the aggressive nature of GBM.

## Introduction

Isocitrate dehydrogenase (IDH)-wildtype glioblastoma (GBM) is the most common and malignant astrocytic glioma, accounting for approximately 90% of all GBM cases, and typically affects adults^1^. Although the tumor is generally treated with surgical resection, chemotherapy, and radiation, the overall survival (OS) of IDH-wildtype GBM is approximately 22 months from the initial tumor diagnosis^2–4^. Moreover, a retrospective review of GBM patients at MDACC between 2006 and 2012, which identified 330 recurrent GBM patients, reported that the median OS for trials at the first recurrence was 9.8 months for IDH1-wildtype GBM, while that for IDH1-mutant GBM was 19.32 months^3^. Therefore, it is important to understand the molecular basis of IDH1-wildtype GBM, which causes heterogeneity and aggressiveness.

Several previous studies evaluated the expression and role of branched-chain amino acid trasaminase1 (BCAT1) in IDH1-wildtype glioma^5–9^. BCAT1 is a cytosolic enzyme that catalyzes the transformation of branched-chain L-amino acids (BCAA) into branched-chain a-ketoacids (BCKA), with concomitant conversion of a-KG to glutamate^5,10–12^.

Tönjes *et al*.^6^ found that IDH-wildtype GBM has increased expression of BCAT1, and they showed that this enzyme is necessary for tumor growth and disease progression. Another study reported^10^ that expression of BCAT1 was significantly increased in IDH1-wildtype glioma cells compared with IDH1-mutant glioma cells, which was investigated by hyperpolarized ^13^C magnetic resonance spectroscopy (MRS). In addition, previous studies have shown that BCAT1 could serve as a novel target for GBM treatment^13^. Therefore, noninvasive assessment of BCAT1 activity could help with the diagnosis and monitoring of IDH1-wildtype GBM.

In this regard, a previous study demonstrated that bevacizumab resistance increased with the expression of BCAT1 in IDH1-wildtype rat GBM, which was assessed by dynamic contrast susceptibility (DSC) perfusion MRI. Moreover, normalized cerebral blood volume (nCBV) could be a surrogate imaging biomarker for the prediction of anti-angiogenic treatment in IDH1-wildtype rat GBM^9^. These results raise the question of which MR imaging parameters can be used as representative imaging biomarkers for predicting BCAT1 expression in IDH1-wildtype GBM patients.

Advanced imaging analysis allows for noninvasive, three-dimensional and quantitative characterization of neoplasms^14,15^ and has great potential for guiding therapy by providing a comprehensive view of the entire tumor^16,17^. Moreover, the results of several studies that used Cancer Genome Atlas (TCGA) GBM data demonstrated that MR imaging findings can be combined with gene expression, genetic alterations, and patient survival data^18–25^. Therefore, in this study, we sought to assess the MR imaging features and prognosis related to the expression level of BCAT1 in IDH1-wildtype GBM.

## Results

The baseline epidemiologic and molecular characteristics are shown in Table 1. In brief, 39 male and 30 female patients were included in the present study. The average age was 53.8 ± 12.9; the IDH1 or IDH2 mutation was observed in 9 (13%) and 2 (3%) patients, respectively, and 34 (49.2%) patients showed MGMT promoter methylation status.

**Table 1:** Clinical Characteristics of the Patients.

Parameter	
No. of patients	69
Mean age (y)	53.8 ± 12.9
Sex	
Male	39 (56.5)
Female	30 (43.5)
Mean radiation dose (Gy)	56.11 ± 9.42
IDH mutation status	
IDH1-wildtype	60 (87)
IDH1-mutant	9 (13)
IDH2-wildtype	67 (97)
IDH2-mutant	2 (3)
MGMT promoter methylation status	
Methylated	34 (49.2)
Unmethylated	35 (50.8)

Note. - Except where indicated, data are given as the numbers of patients with percentages in parentheses.

### BCAT1 expression status in GBM

**Figure 1:**
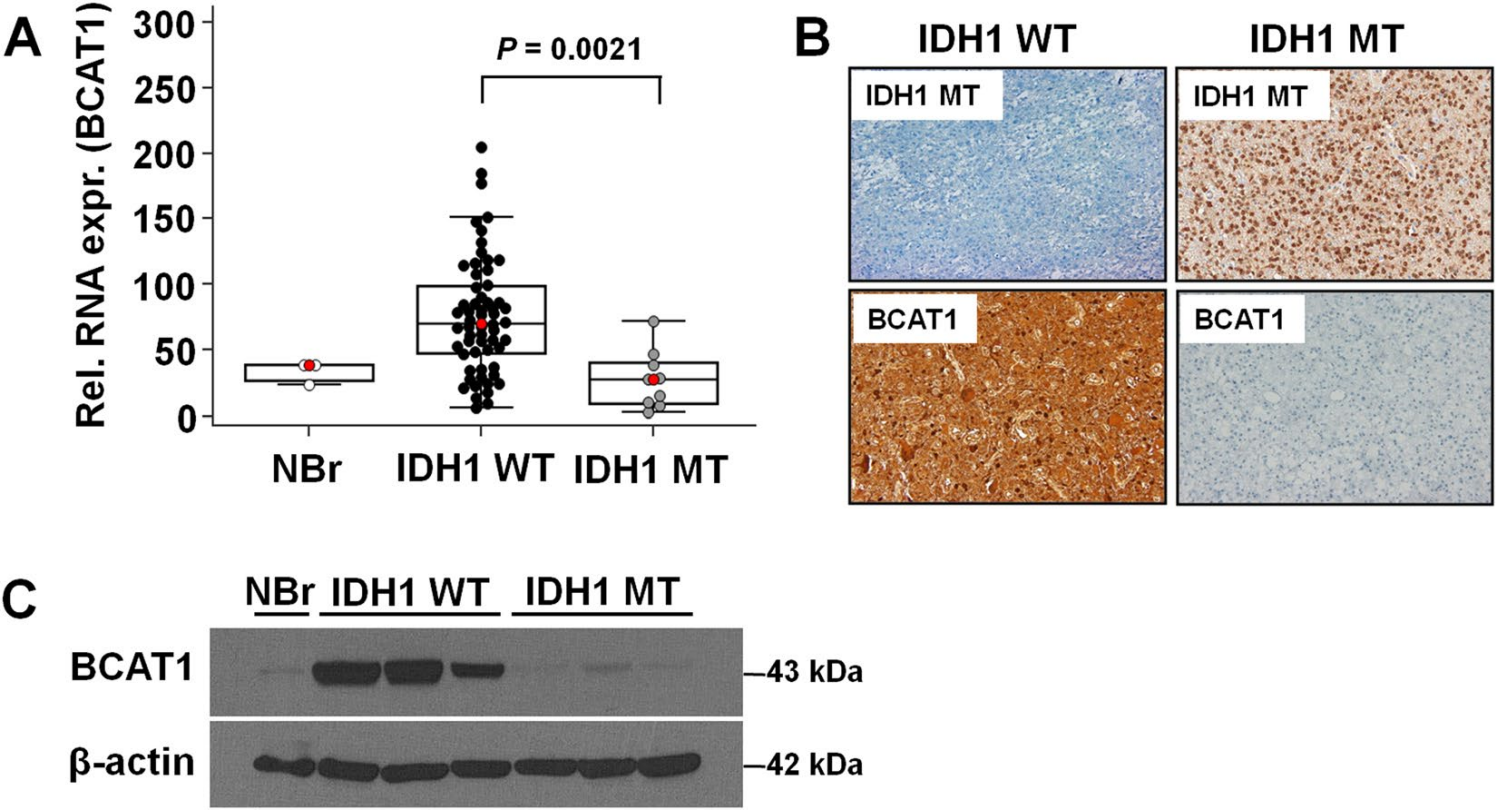
(**A**) qRT-PCR analysis revealed that the BCAT1 messenger RNA level is significantly increased in IDH1-wildtype patients compared with IDH1-mutant patients (*P* = 0.0021). (**B**,**C**) Immunohistochemistry and western blotting show that the BCAT1 protein expression level is higher in IDH1-wildtype patients compared with IDH1-mutants. NBr = Normal Brain, WT = wildtype, MT = mutant. We didn’t use cropping for western blot. (See Supplementary Fig. S1 for full-length blots).

The BCAT1 RNA expression levels were significantly higher in IDH1-wildtype GBMs than IDH1-mutant tumors (*P* = 0.0021) (Fig. 1 and Table 2). Similar to these RNA expression results, we used immunohistochemical staining and western blotting to observe that the BCAT1 protein expression was also higher in IDH1-wildtype patients.

**Table 2:** BCAT1 expression level with respect to the IDH status.

IDH1 status	BCAT1 (Rel. RNA expression level)
IDH1-wildtype (*n* = 60)	75.8662 ± 44.2196
IDH1-mutant (*n* = 9)	27.6722 ± 22.2112
IDH2-wildtype (*n* = 67)	70.2916 ± 45.2910
IDH2-mutant (*n* = 2)	45.74 ± 30.6036

### Correlation of quantitative image features with BCAT1 expression and MGMT promoter methylation

**Figure 2:**
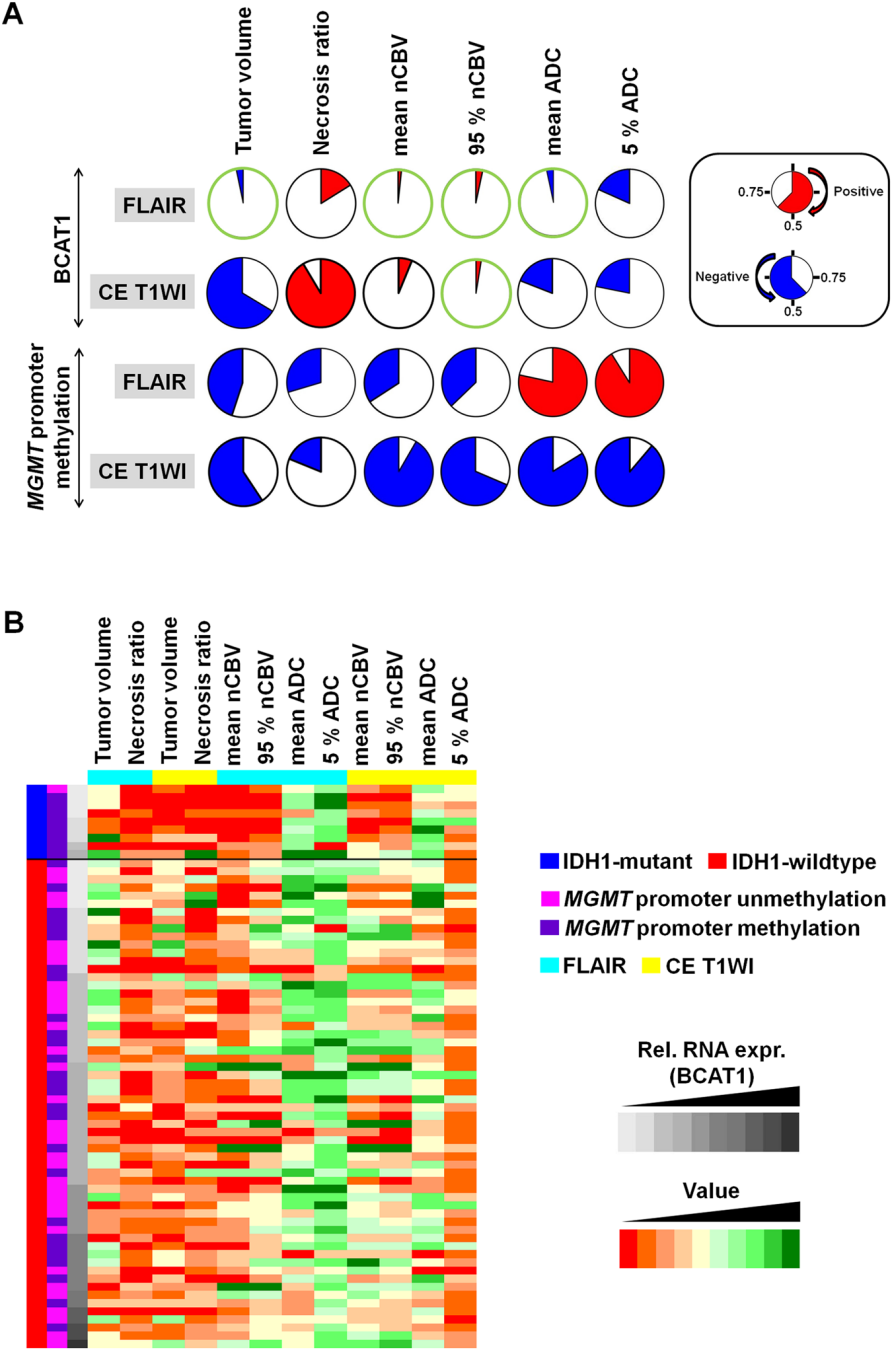
(**A**) The pie charts derived by the relationship between the quantitative MR parameters and BCAT1 expression level in GBM depict the *P* values for each pairwise correlation. The *P* values are marked by circle filling and color intensity; *R* *ed* = positive correlation and *blue* = negative correlation. Correlations determined to be statistically significant are marked with a green circle. The BCAT1 expression level was significantly correlated with the mean and 95^th^ percentile-normalized CBV as well as the mean ADC based on FLAIR images (*P* < 0.05). In addition, the 95^th^ percentile-normalized CBV from CE T1WI also had a significant correlation with the BCAT1 expression level (*P* = 0.0255). (**B**) Heat map of the evaluated MR imaging parameters associated with BCAT1 expression in GBM patients. The values of the MR parameters are represented by color; orange indicates a value less than and green indicates a value greater than the median value for the given parameter set. According to the increase in the BCAT1 expression level, the heat map shows an increase in the CBV and decrease in ADC based on FLAIR as well as a decrease in the ADC based on CE T1WI. *Blue* = IDH-mutant group, *Red* = IDH-wildtype group, *FLAIR* = fluid-attenuated inversion recovery, *CE* = contrast-enhancing, *CBV* = cerebral blood volume, and *ADC* = apparent diffusion coefficient.

The correlation analysis between the BCAT1 expression level or MGMT promoter methylation status and quantitative values from tumor volumetrics and nCBV and ADC histograms were performed. (Fig. 2 and Supplementary Figs S2–S4) Among the volumetrics parameters, only the tumor volume based on FLAIR images had a significant correlation with the BCAT1 expression level (*r* = −0.2579, *P* = 0.0324). From the tumor extents based on FLAIR images, the BCAT1 expression level was significantly correlated with the mean nCBV, 95% nCBV, and mean ADC (*r* = 0.2879, 0.2567 and −0.2562, respectively; in all, *P* < 0.05). In addition, the 95% nCBV value from the enhancing area based on CE T1WI also had a significant correlation with the BCAT1 expression level (*r = *0.2689, *P* = 0.0255). Multiple regression analysis revealed that the only tumor volume (*P* = 0.0294) and mean ADC value (*P* = 0.0407) based on FLAIR images were significantly correlated with BCAT1 expression level independently with IDH1 mutation status. None of the quantitative imaging parameters had a significant correlation with the MGMT promoter methylation status.

In IDH1-wildtype group, the tumor volume (*r* = −0.3473, *P* = 0.0066) and mean ADC value (*r* = −0.3059, *P* = 0.0175) based on FLAIR images were also significantly correlated with BCAT1 expression level, while no imaging parameters had significant correlation with BCAT1 expression level in IDH1-mutant group (Supplementary Table S2).

**Figure 3:**
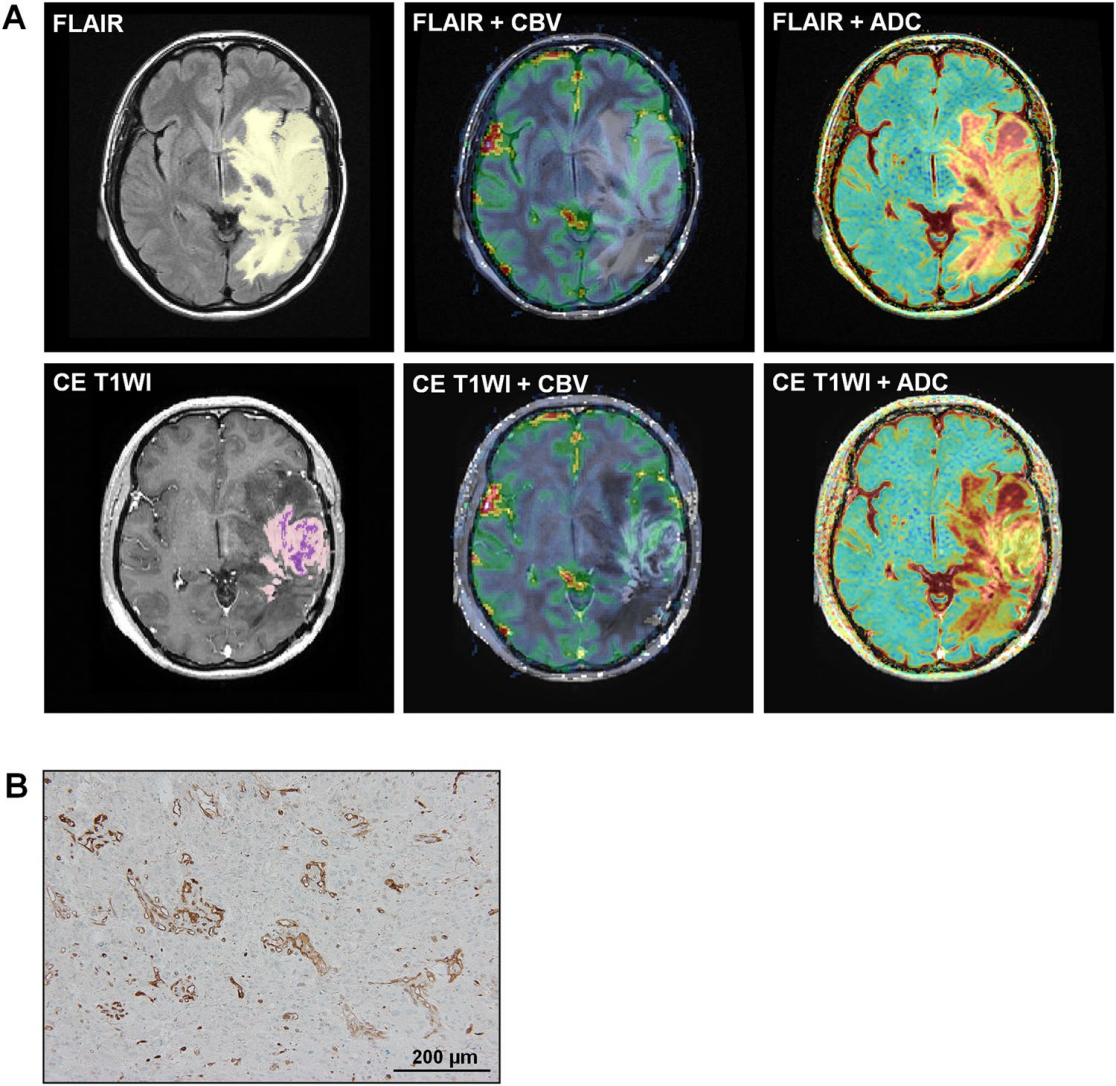
Images in a 43-year-old woman diagnosed with GBM and a low level (17.63) of BCAT1 expression. (**A**) The tumor showed low nCBV and high ADC values for both FLAIR (Top line, mean nCBV: 1.53, mean ADC: 1481.18 × 10^−6^ mm^2^/sec) and contrast enhanced T1W1 (Bottom line, mean CBV: 2.98, mean ADC: 1555.15 × 10^−6^ mm^2^/sec). The yellow and light purple ROIs indicate the entire tumor areas on FLAIR and CE T1WI, respectively, and the deep purple ROI represents the necrotic portion within the tumor on CE T1WI. (**B**) Histology showed a low level of BCAT1 expression.

**Figure 4:**
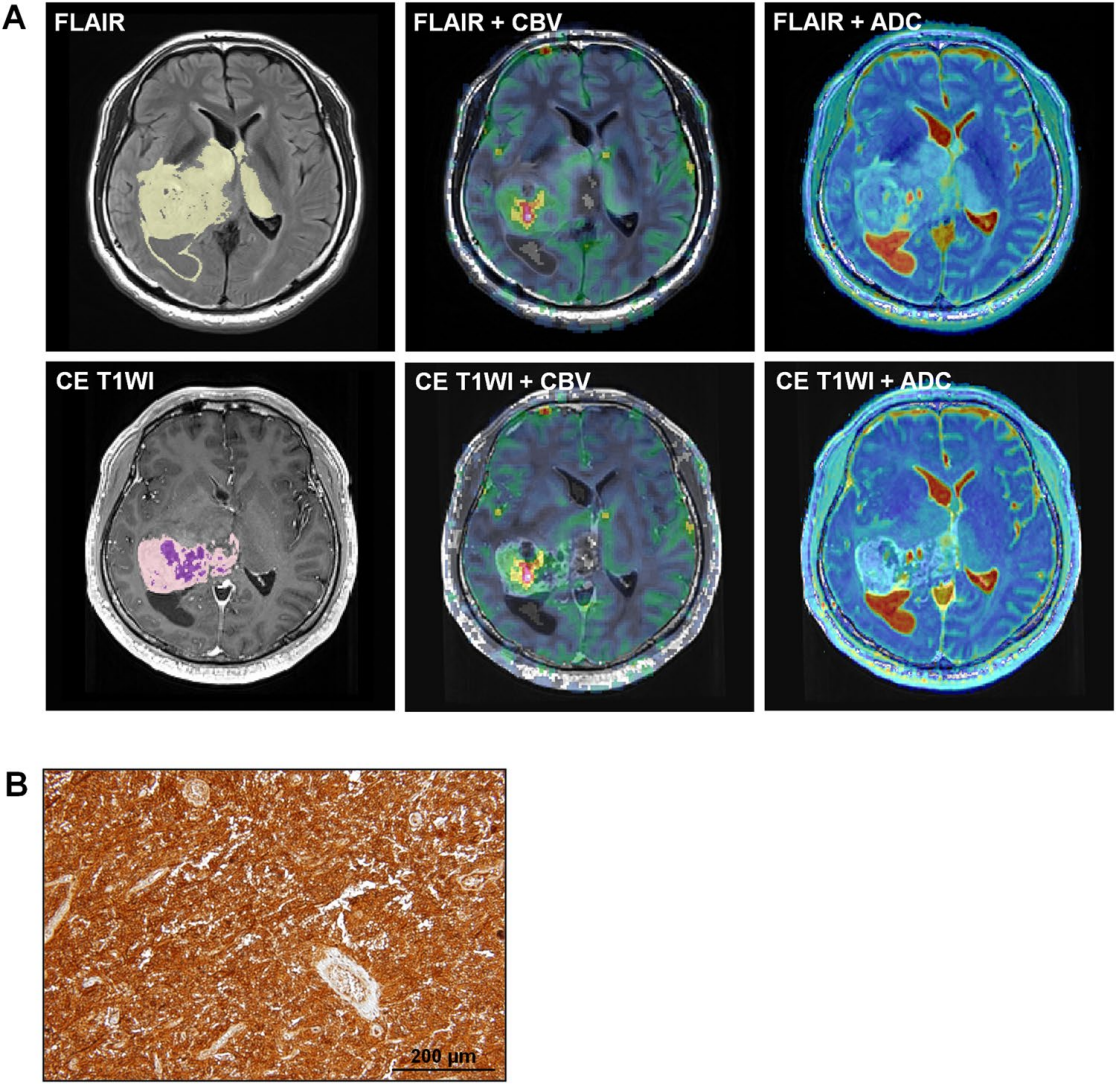
Images in a 42-year-old man diagnosed with GBM and a high level (204.9) of BCAT1 expression. (**A**) The tumor showed high nCBV and low ADC values from both FLAIR (Top line, mean nCBV: 6.86, mean ADC: 1211.26 × 10^−6^ mm^2^/sec) and contrast enhanced T1W1 (Bottom line, mean nCBV: 11.00, mean ADC: 1225.30 × 10^−6^ mm^2^/sec). The yellow and light purple ROIs indicate the entire tumor areas on FLAIR and CE T1WI, respectively, and the deep purple ROI represents the necrotic portion within the tumor on CE T1WI. (**B**) Histology showed a low level of BCAT1 expression.

The representative cases reveal that the MR imaging features were correlated with BCAT1 expression of IDH1-wildtype GBMs. Tumors with high expression of BCAT1 had a high nCBV and low ADC value, and vice versa (Figs 3 and 4).

### Reproducibility analysis

Interobsever reproducibility study revealed that intraclass coefficients (ICCs) of each quantitative parameter based on both FALIR and CE T1WI images showed excellent reproducibility (0.9782–0.9988) (Supplementary Table S2).

### Survival analysis

**Figure 5:**
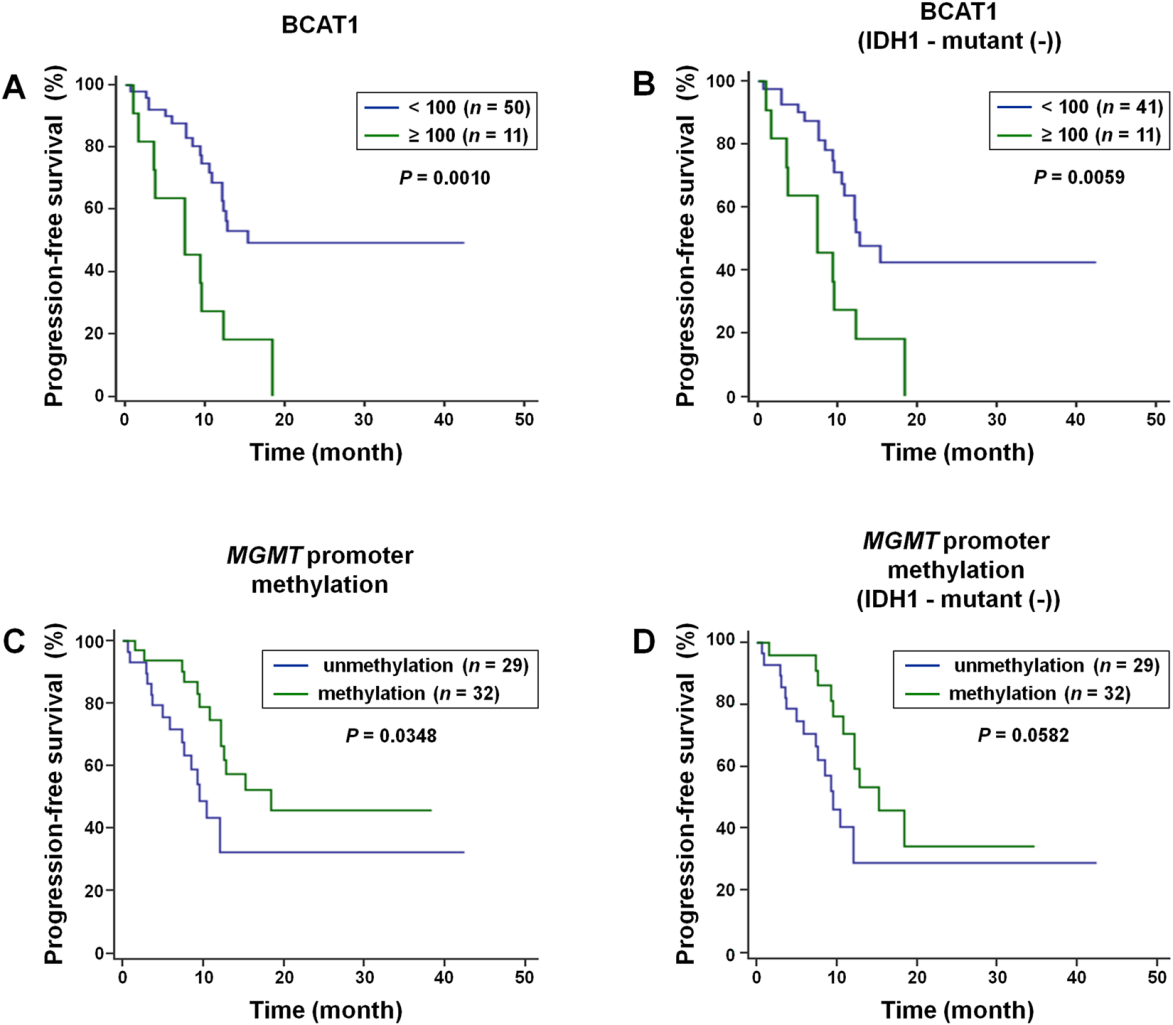
Kaplan-Meier estimates of progression-free survival according to the BCAT1 expression level and MGMT promoter methylation status. (**A**) In all patients, there was a significant difference in PFS between low and high BCAT1 expression tumors (median, 15.2 [95% CI, 12.1–15.2] vs 7.4 [95% CI, 3.5–12.2] months; *P* = 0.0010, logrank test). (**B**) The patients with IDH1-wildtype GBMs also showed a significant difference in PFS between low and high BCAT1expression tumors (median, 12.8 [95% CI, 10.8–15.2] vs 7.4 [95% CI, 3.5–12.2] months; *P* = 0.0059, logrank test). (**C**,**D**) In terms of the MGMT promoter methylation status, PFS was significantly longer in the positive tumors than negative ones (median, 18.4 [95% CI, 12.2–18.4] vs 9.5 [95% CI, 7.6–12.1] months; *P* = 0.0348, logrank test) in all patients, but, among patients with IDH1-wildtype GBMs, there was no significant difference between patients with positive and negative MGMT promoter methylation (median, 15.2 [95% CI, 12.2–18.4] vs 9.5 [95% CI, 7.6–12.1] months; *P* = 0.0582, logrank test).

We generated Kaplan–Meier curves to compare PFS according to the BCAT1 expression level (<100 vs ≥100) and MGMT promoter methylation status (Fig. 5). In all patients, there was a significant difference in PFS between low and high BCAT1 expression tumors (median, 15.2 [95% CI, 12.1–15.2] vs 7.4 [95% CI, 3.5–12.2] months; *P* = 0.0010, logrank test). Patients with IDH1-wildtype GBMs also had a significant difference in PFS between low and high BCAT1 expression tumors (median, 12.8 [95% CI, 10.8–15.2] vs 7.4 [95% CI, 3.5–12.2] months; *P* = 0.0059, logrank test). In terms of the MGMT promoter methylation status, PFS was significantly higher in positive tumors than negative tumors (median, 18.4 [95% CI, 12.2–18.4] vs 9.5 [95% CI, 7.6–12.1] months; *P* = 0.0348, logrank test) in all patients. However, among patients with IDH1-wildtype GBMs, there was no significant difference between patients with positive and negative MGMT promoter methylation (median, 15.2 [95% CI, 12.2–18.4] vs 9.5 [95% CI, 7.6–12.1] months; *P* = 0.0582, logrank test).

When we performed a Cox multivariate analysis, we found that a BCAT1 expression level ≥100 (*P* = 0.0001) and MGMT promoter methylation (*P* = 0.0032) emerged as independent prognostic factors for PFS (Table 3).

**Table 3:** BCAT1 expression level with respect to the IDH status.

Covariate	b	SE	*P*	Exp(b)	95% CI of Exp(b)
BCAT1 expression level (≥100)	1.702	0.4448	0.0001	5.4847	2.2935–13.1162
MGMT	−1.2664	0.4289	0.0032	0.2819	0.1216–0.6533

Abbreviations: *b* = coefficient estimates, *95% CI =* 95% confidence interval, *Exp(b)* = hazard ratio value, *SE* = standard error for coefficient estimates b.

## Discussion

In this study, we assessed the quantitative MR imaging features and prognosis related to the BCAT1 expression level in GBM patients. We found significant positive correlations between the BCAT1 expression level and MR imaging features, including the mean nCBV and 95% nCBV from the tumor extents based on FLAIR images and 95% nCBV value from the enhancing area based on CE T1WI. In all patients and IDH1-wildtype group, the tumor volume and mean ADC value based of FLAIR images had a significant negative correlation with BCAT1 expression, which was observed independently with IDH1 mutation status. However, none of the quantitative imaging parameters had a significant correlation with the MGMT promoter methylation status. In terms of survival analysis, a longer PFS was observed in GBM patients with low BCAT1 expression than those with high expression, regardless of the IDH1 mutation status, while there was no significant difference in PFS according to the MGMT promoter methylation status in patients with IDH1-wildtype GBMs. In addition, the BCAT1 expression level had a higher hazard ratio than the MGMT promoter methylation status.

According to the previous clinical study by Panosyan *et al*., BCAT1 enzymes appear to be associated with clinical aggressiveness and recurrence of malignant gliomas as well as with progression of newly diagnosed GBMs. In addition, IDH1-wildtype gliomas have increased expression of this gene^7^. In our study, we found that the aggressive nature of GBMs with a high expression level of BCAT1 was also correlated with the MR imaging features as well as the clinical prognosis. In terms of the physiologic imaging parameters of GBM, we measured the nCBV and ADC values, which reflect tumor angiogenesis and cellularity, respectively^26,27^. These imaging parameters were used to evaluate tumor characteristics, such as invasiveness, aggressiveness and prognosis, especially in GBM. Several studies have shown that the hemodynamic parameter CBV from DSC perfusion MR imaging is an important prognostic imaging biomarker that provides useful prognostic information in GBM patients^21,28^. An increased CBV in contrast-enhancing lesions is associated with an increased risk of death, and a high CBV in non-enhancing lesions is also associated with poor survival^29^. The ADC value is an important imaging biomarker that is related to tumor invasion and aggressiveness^30^. The ADC of GBM, normalized by the ADC of white matter, demonstrated an inverse correlation between the normalized ADC and histopathologic features of aggressiveness^31^. The aggressive nature was related to intracellular BCAT1 metabolism of GBM and can be interpreted by using the measurement of CBV and ADC. In addition, mean ADC value based on FLAIR imaging had significant association with BCAT1 expression, regardless of IDH1 mutation status, which seems to be more closely associated with BCAT1-related GBM aggressiveness than CBV values.

Interestingly, the GBMs included in our study demonstrated a negative correlation between the BCAT1 expression level and volume based on FLAIR images independently with IDH1 mutation status. Because the T2 hyperintense area of tumor represents peritumoral edema and tumor cell infiltration, larger T2 hyperintense areas of GBMs may suggest slowly-growing tumors, resulting in a larger overall tumor volume, which is consistent with previous studies^32,33^. Therefore, GBMs with a low expression level of BCAT1 tend to have a slow growing nature, resulting in a relatively large tumor volume based on FLAIR images.

MGMT is a key gene that encodes a protein that repairs DNA, while methylation suppresses the DNA repair activity, including DNA in the tumor that is actively dividing. Therefore, GBMs with MGMT promoter methylation can be expected to respond better to an alkylating agent, such as temozolomide^34^. In addition, MGMT methylation may be considered to be a predictive biomarker for a patient’s desirable response to radiation therapy. Several reports in the literature indicate that MGMT promoter methylation is associated with longer survival^35^. In our study, the BCAT1 expression level was better correlated with prognosis and imaging features than the MGMT methylation status. According to a previous study, both PFS and overall survival are adversely affected by higher levels of GBM expression as well as by high levels of the protein detected by IHC in HGGs^36^. The BCAT1 expression level is a clinically useful prognostic biomarker for GBM patients, and its activity can be monitored with imaging features.

We recognize several limitations in this study. First, the retrospective design of this study with the small sample size may have introduced inherent selection bias. Second, we used tumor samples that were randomly obtained from main masses in each patient, which cannot precisely evaluate the tumor heterogeneity. A future prospective study is needed to address this issue. Third, we did not analyze the metabolism alteration related to BCAT1 expression in GBM cells, which can explain the associated imaging features of GBM. This issue also requires future study.

In conclusion, we found that a high BCAT1 expression level is correlated with a high CBV, low ADC value, and poor prognosis. BCAT1 expression is likely related to the aggressive nature in GBM, which seems to be crucial in selecting tailored treatment for IDH1-wildtype GBM patients.

## Materials and Methods

This retrospective human study was approved by the institutional review board of Seoul National University Hospital, which waived the requirement for obtaining informed consent.

### Patient enrollment

Between August 2012 and December 2015, 258 patients who were initially diagnosed with GBM at our institution were consecutively recruited. The inclusion criteria were as follows: the patient (a) had a histopathologic diagnosis of GBM without other cell components based on the World Health Organization criteria; (b) underwent conventional, diffusion-weighted imaging (DWI) and DSC perfusion MR imaging 24–48 hours before surgery; (c) had available tumor samples in the brain tumor bank of our institute; and (d) underwent the standard treatment of near-total resection, concomitant chemoradiotherapy (CCRT) and adjuvant temozolomide medication. Based on these inclusion criteria, 69 patients were included in our study. All tumor samples used in this study were snap-frozen in liquid nitrogen as soon as possible during surgery and stored at −80 °C.

### RNA isolation and real-time PCR

The total RNA of each tissue sample was isolated using a QIAquick RNeasy Mini kit (Qiagen) according to the manufacturer’s instructions, and the quality of the RNA was verified with an Agilent 2100 Bioanalyzer (Agilent Technologies). Reverse transcription was performed with RevertAid H Minus Reverse Transcriptase (Thermo). Briefly, reverse transcription was performed in a volume of 100 μl with 2.0 μg of RNA; 15 pmol of oligo deoxythymidine primer; 20 µl of 5 × RT Buffer; and 20 μl each of 2.5 mM dNTP mix, RNase inhibitor, and reverse transcriptase. The RT conditions were as follows: 10 minutes at 65 °C, 60 minutes at 42 °C, 10 minutes at 25 °C, and 10 minutes at 70 °C.

Real-time PCR was performed in a Rotor-Genes Q cycler machine (Qiagen) using a Rotor-Genes SYBR Green PCR kit (Qiagen) according to the manufacturer’s instructions in a total volume of 20 µl. The cycling conditions for the BCAT1 and GAPDH genes were 10 minutes at 95 °C with 40 cycles of 10 seconds at 95 °C, 15 seconds at optimal Tm, and 20 seconds at 72 °C. The sequences of the primers were as follows:

BCAT1 5′-caactatggagaatggtcctaagct-3′ and 5′-tgtccagtcgctctcttctcttc-3′ and GAPDH 5′-ggcattgctctcaatgacaa-3′ and 5′-atgtaggccatgaggtccac-3′. To correlate the threshold (Ct) values from the amplification plots to the copy number, a standard curve was generated and a non-template control was run with every assay. All samples were run in duplicate, and the average value was used.

### Western blot

The protein levels were evaluated by western blot analysis. Tissues were lysed in ice-cold lysis buffer, and the concentration of protein was evaluated with the bicinchoninic acid method (Pierce Biotechnology). Approximately 30 μg of protein was loaded in each lane of a polyacrylamide denaturing gel for electrophoresis. After electrophoresis, the protein was transferred to nitrocellulose membranes for blotting. We used a mouse monoclonal antibody to human BCAT1 (OriGene) and a rabbit polyclonal antibody to β-actin (Abcam). Primary antibodies were detected by horseradish peroxidase-conjugated antibodies (Santa Cruz Biotechnology).

### Immunohistochemical staining

Immunohistochemical staining was performed using formalin-fixed paraffin-embedded tumor blocks. Briefly, 4-µm thick tissue sections were deparaffinized in xylene and hydrated by immersing them in a series of graded ethanol. Antigen retrieval was performed in a microwave by placing the sections in epitope retrieval solution (0.01 M citrate buffer, pH 6.0) for 20 minutes; endogenous peroxidase was inhibited by immersing the sections in 0.3% hydrogen peroxide for 10 minutes. Sections were then incubated with the primary mouse monoclonal antibody to BCAT1 (BD Biosciences) and mouse monoclonal antibody to human IDH1 R132H (Dianova) in Dako REAL antibody diluent (Dako). Staining to detect the bound antibody was evaluated by DAB.

### Identification of O^6^-methylguanine methyltransferase (MGMT) methylation and IDH mutation

MGMT methylation-specific PCR (MSP) using an EZ DNA methylation-Gold Kit (Zymo Research, Orange County, CA, USA) was used to evaluate the methylation status of the MGMT promoter. Sanger sequencing was used to analyze the frequency of IDH1 and IDH2 mutations.

### MRI protocol

All patients underwent conventional, DWI and DSC perfusion MRI using a 3T scanner (Verio; Siemens Healthcare Sector) with a 32-channel head coil. The conventional MRI included T1-weighted imaging (T1WI), which included transverse spin-echo imaging with before and after contrast enhancement or multi-planar reconstructed transverse, coronal imaging with a sagittal three-dimensional magnetization prepared rapid acquisition gradient echo (3D-MPRAGE) sequence with before and after contrast enhancement, as well as transverse T2-weighted imaging (T2WI) with turbo spin-echo sequences and FLAIR images. Contrast-enhanced (CE) T1WI was acquired after intravenous administration of gadobutrol (Gadovist^®^, Bayer Schering Pharma) at a concentration of 0.1 mmol per kilogram (mmol/kg) of body weight. Transverse spin-echo T1-weighted imaging was performed with the following parameters: repetition time (TR), 558 ms; echo time (TE), 9.8 ms; flip angle (FA), 70°; matrix, 384 × 187; field-of-view (FOV), 175 × 220 mm; section thickness, 5 mm; and number of excitations (NEX), 1. We obtained the 3D-MPRAGE sequences using the following parameters: TR, 1500 ms; TE, 1.9 ms; FA, 9°; matrix, 256 × 232; FOV, 220 × 250; section thickness, 1 mm; and NEX, 1. The parameters of the transverse T2-weighted imaging were as follows: TR, 5160 ms; TE, 91 ms; FA, 124–130°; matrix, 640 × 510–580; FOV, 175–199 × 220; section thickness, 5 mm; and NEX, 3. The parameters for transverse FLAIR were a TR of 9000 ms, TE of 97 ms, TI of 2500 ms, FA of 130°, matrix of 384 × 348, FOV of 199 × 220, section thickness of 5 mm and NEX of 1.

DWI was performed with a single-shot spin-echo echo-planar imaging (EPI) sequence in the axial plane before injection of contrast material with b-values of 0 and 1000 sec/mm^2^, a TR of 6300 ms, TE of 92 ms, FA of 180°, matrix of 240 × 240, FOV of 240 × 240, section thickness of 3 mm and NEX of 3. DWI was acquired in three orthogonal directions and combined into a trace image. Using these data, ADC maps were calculated on a voxel-by-voxel basis with the software that was incorporated into the MRI unit.

Transverse DSC perfusion MRI was performed with single-shot gradient-echo echo-planar sequences during the intravenous administration of gadobutrol at a concentration of 0.1 mmol/kg of body weight at a rate of 4 mL/sec using a power injector (Spectris; Medrad). A 30-mL bolus injection of saline was administered at the same injection rate. For each section, 60 images were acquired at intervals equal to the TR. The parameters were as follows: TR, 1500 ms; TE, 30 ms; FA, 90°; matrix, 128 × 128; section thickness, 5 mm; intersection gap, 1 mm; FOV, 240 × 240 mm; sections, 15–20; voxel size, 1.875 × 1.875 × 5 mm^3^; pixel bandwidth, 1563 Hz; and total acquisition time, 1 minute 30 seconds.

### Image post-processing and data analysis

The conventional MR images, ADC maps, and DSC PWI were digitally transferred from the picture archiving and communication system workstation to a personal computer for further analysis. The relative CBV (rCBV) was obtained with a dedicated software package (nordicICE; Nordic Imaging Lab, Bergen, Norway) that applied an established tracer kinetic model to the first-pass data^37,38^. First, realignment was performed to minimize patient motion during the dynamic scans. A gamma-variate function, which approximates the first-pass response as it would appear in the absence of recirculation, was used to fit the 1/T2* curves to reduce the effects of recirculation. To reduce the contrast agent leakage effects, the dynamic curves were mathematically corrected^39^. After elimination of recirculation and leakage of the contrast agent, rCBV was computed with numeric integration of the curve. To minimize variances in rCBV in an individual patient, the pixel-based rCBV maps were normalized by dividing every rCBV value in a specific section by the rCBV value in the unaffected white matter, which was used for nCBV map^40^.

Co-registrations between the structural images (e.g., FLAIR images and CE T1WI) and the nCBV and ADC maps were performed based on the geometric information stored in the respective data sets using a dedicated software package (nordicICE). The differences in the slice thicknesses between images were automatically corrected by re-slicing and co-registration, which were based on the underlying structural images. The nCBV and ADC maps were displayed as color overlays on the both FLAIR images and CE T1WI.

One neuroradiologist (with 16 years of brain MR imaging experience), who was blinded to the clinical data, drew polygonal region of interests (ROIs) that contained all of the enhancing lesions in each section of the co-registered images. Areas of necrosis, hemorrhage, or non-tumor macro-vessels that were evident on the CE T1WI were excluded from the ROIs. Then, the ROIs of T2 high SI lesions, regardless of contrast enhancement, were defined on each transverse FLAIR image, avoiding cystic, necrotic regions and macrovessels. Because the ROI placement was conducted on the nCBV and ADC map co-registered with structural images, the margin of the lesions could be defined with confidence. The entire volume of the contrast-enhanced lesions, T2 high SI lesions, and necrosis, which was defined as a hypointense area without contrast enhancement on CE T1WI within the mass on the FLAIR images, was calculated. The volumes and ADC values were expressed in units of mL and ×10^−6^ mm^2^/sec, respectively.

The data acquired from each section were summed to derive the voxel-by-voxel ADCs and nCBVs for the entire tumor extent based on both CE T1WI and FLAIR images with nordic ICE. The ADC and nCBV histograms were plotted with ADC and nCBV on the respective x-axis with bin sizes of 3 × 10^−5^ mm^2^/sec and 0.1, respectively, whereas the y-axis was expressed as the percentage of the total lesion volume by dividing the frequency in each bin by the total number of analyzed voxels. For further quantitative analysis, the cumulative number of observations in all bins up to the specified bin was mapped on the y-axis as a percentage in the cumulative histograms. The 5th percentile point for ADC (5% ADC) and 95th percentile point for nCBV (95% nCBV) were derived (the Xth percentile point is the point at which X% of the voxel values that form the histogram are found to the left of the histogram)^41,42^.

One more neuroradiologist (with 8 years of brain MR imaging experience), who was also blinded to the clinical data, repeated drawing polygonal ROIs on both CE T1WI and FLAIR images to measure all imaging parameters described above. And then, we calculated ICCs of each quantitative parameter to evaluate the reproducibility.

### Statistical analysis

All statistical analyses were performed using two commercial software programs (MedCalc version 13.1.0.0, MedCalc Software). A *P* value < 0.05 was considered statistically significant. Kolmogorov-Smirnov’s test was used to determine whether the non-categorical variables were normally distributed. Non-parametric data are presented as the median and interquartile range (IQR, range from the 25th to the 75th percentile), and parametric data are shown as the mean ± standard deviation. Based on the results of Kolmogorov-Smirnov’s test, unpaired Student’s t-test or a Mann-Whitney U-test was performed, as appropriate, to compare the values between two groups. Pearson correlation analysis and Spearman rank correlation test were performed for the correlation between the BCAT1 expression level and quantitative imaging parameters in parametric and non-parametric data, respectively. Multiple regression analysis was conducted to determine the correlation between the quantitative imaging parameters and BCAT1 expression level independently with IDH1 mutation status. Interobserver reproducibility was considered as poor (ICC, 0.00–0.20), fair to good (ICC, 0.40–0.75), or excellent (ICC, >0.75)^43^.

Progression-free survival (PFS) was assessed by using the Kaplan-Meier method according to the BCAT1 expression level (<100 vs ≥100) and MGMT promoter methylation status, which were compared using log-rank tests. GBM progression was defined according to the RANO criteria^44^. We only recorded the first progression. PFS was calculated from the date of surgery to that of GBM progression, death, final confirmation of no evidence of disease, or most recent follow-up examination. Patients without an event were censored at the date of the most recent follow-up, regardless of whether they were scheduled for future follow-up or had been lost to follow-up. Eight patients who died due to progression-unrelated conditions (e.g., infarction and infection) were excluded for PFS analysis. Multivariate analysis was performed using the Cox proportional hazards model, which was adjusted for the prognostic factors, including the BCAT1 expression level (<100 vs ≥100) and MGMT promoter methylation status.

All data generated or analysed during this study are included in this published article (and its Supplementary Information files).

## Electronic supplementary material

Supplementary information
