# Supplementary material for: BCAT1 is a New MR Imaging-related Biomarker for Prognosis Prediction in IDH1-wildtype Glioblastoma Patients
Source: Sci Rep. 2017 Dec 18;7:17740. doi: 10.1038/s41598-017-17062-1 (PMC5735129; doi:10.1038/s41598-017-17062-1)
Supplement: Supplementary file 1 — Supplementary information [file 41598_2017_17062_MOESM1_ESM.doc]

**Supplementary Information**

**BCAT1 Is a New MR Imaging-related Biomarker for Prognosis Prediction in IDH1-wildtype Glioblastoma Patients**

Hye Rim Cho^1,2^, Hyejin Jeon^1,2^, Chul-Kee Park^3^, Sung-Hye Park^4^, Kyoung Mi Kang^1^,

Seung Hong Choi^1,2*^

^1^Department of Radiology, Seoul National University Hospital, Seoul, Korea

^2^Center for Nanoparticle Research, Institute for Basic Science (IBS), Seoul, Korea

^3^Department of Neurosurgery, Seoul National University Hospital, Seoul, Korea

^4^Department of Pathology, Seoul National University Hospital, Seoul, Korea

**^*^Corresponding Author:** Seung Hong Choi, MD, PhD

Department of Radiology, Seoul National University College of Medicine

Center for Nanoparticle Research, Institute for Basic Science, and School of Chemical and Biological Engineering, Seoul National University

28, Yongon-dong, Chongno-gu, Seoul, 110-744, Korea

Tel: 82-2-2072-2861; Fax: 82-2-747-7418

E-mail: verocay@snuh.org

**
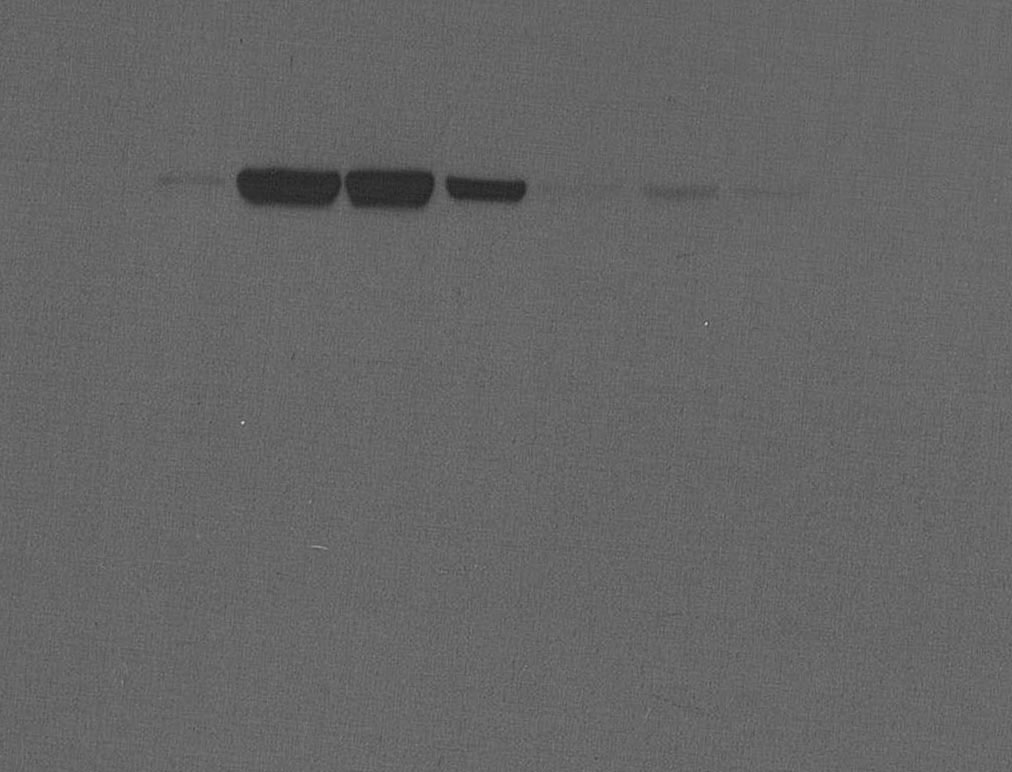
**

**
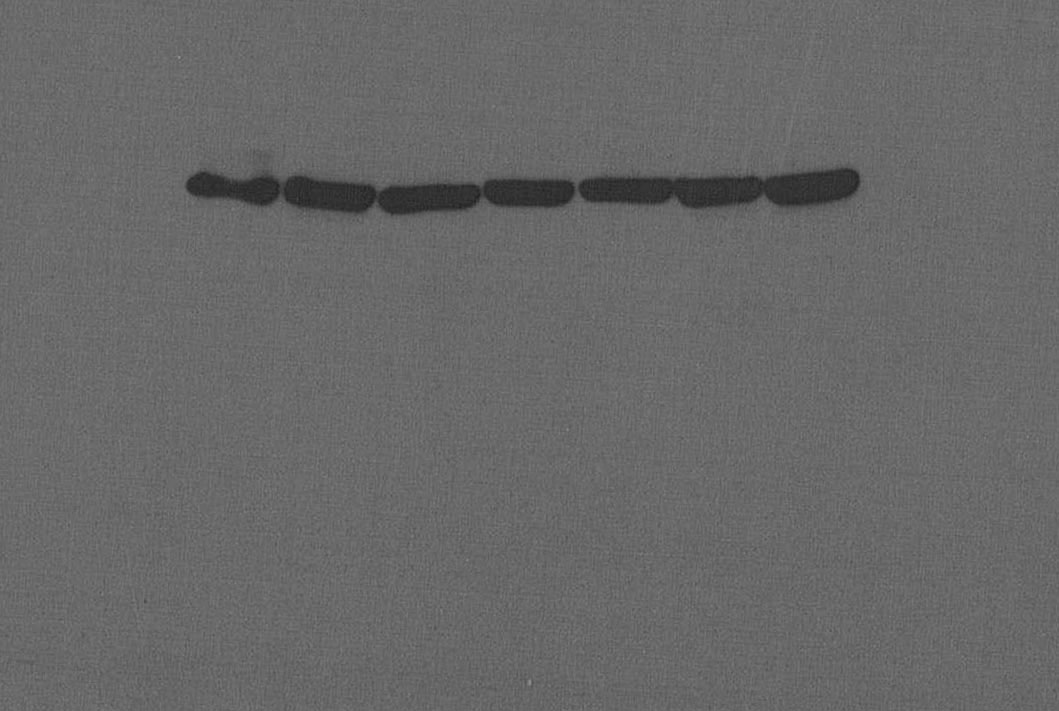
**

**Figure S1.** Full-length blots for western blot. (upper) BCAT1; (lower) β-actin

**
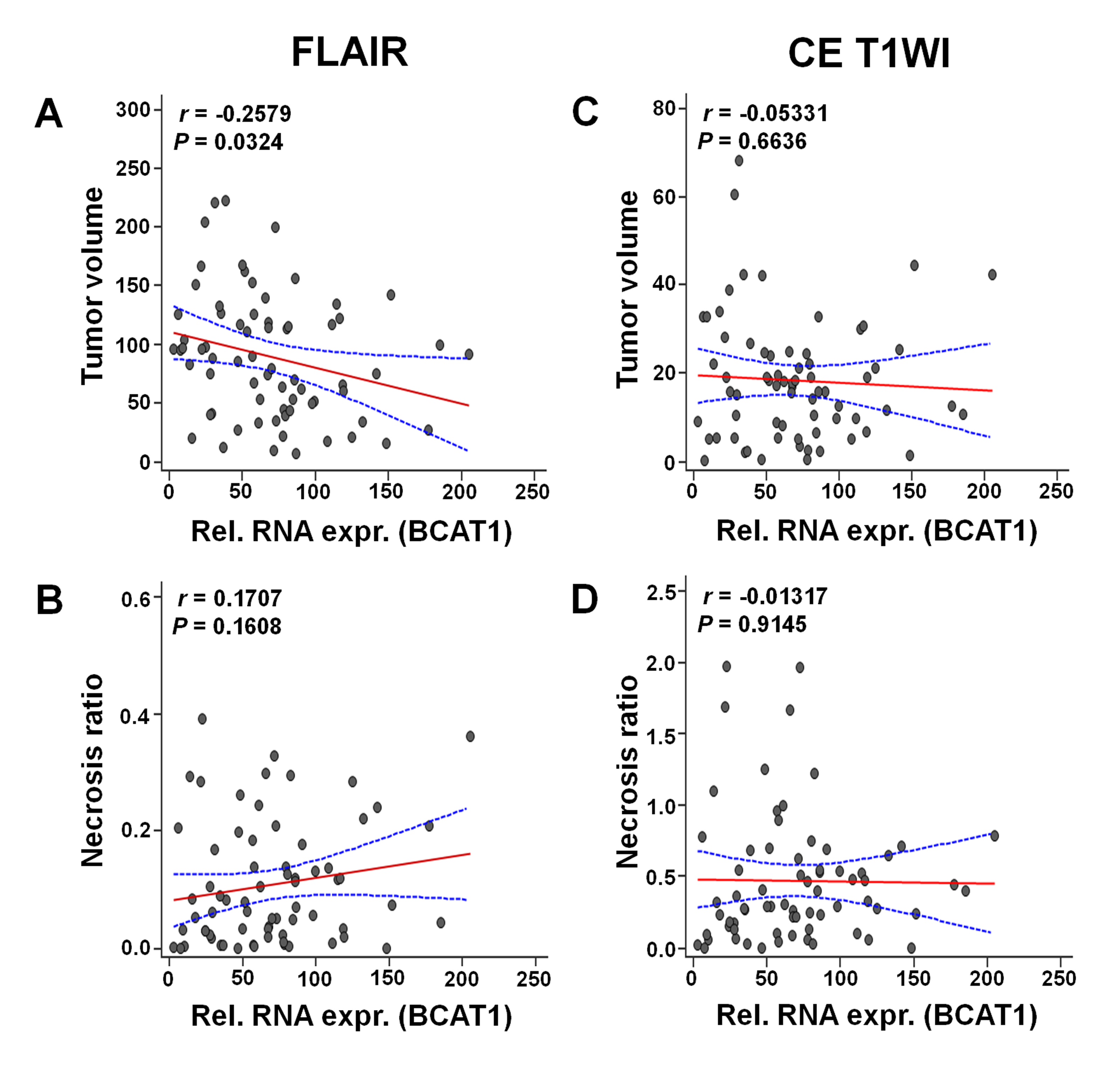
**

**Figure S2.** A scatter diagram and regression line derived by the relationship between the tumor volume / necrosis ratio analyzed from FLAIR / CE image and BCAT1 expression level in GBM. **(A)** FLAIR tumor volume; **(B)** FLAIR necrosis ratio; **(C)** CE T1WI tumor volume; **(D)** CE T1WI necrosis ratio

**
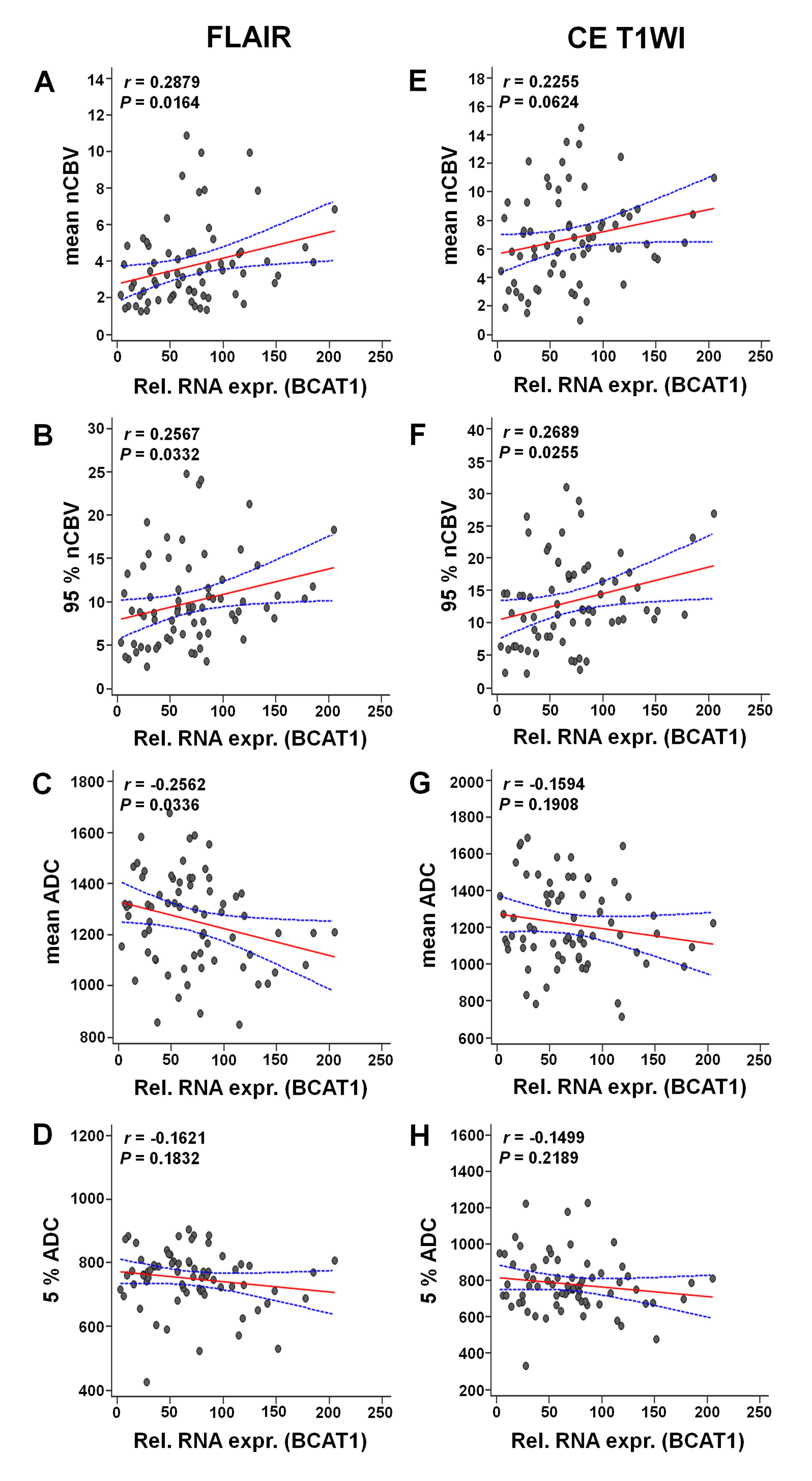
**

**Figure S3.** A scatter diagram and regression line derived by the relationship between FLAIR / CE image parameters and BCAT1 expression level in GBM. **(A)** FLAIR mean nCBV; **(B)** FLAIR 95 % nCBV; **(C)** FLAIR mean ADC; **(D)** FLAIR 5 % ADC; **(E)** CE T1WI mean nCBV; **(F)** CE T1WI 95 % nCBV; **(G)** T1WI CE mean ADC; and **(H)** CE T1WI 5 % ADC

**
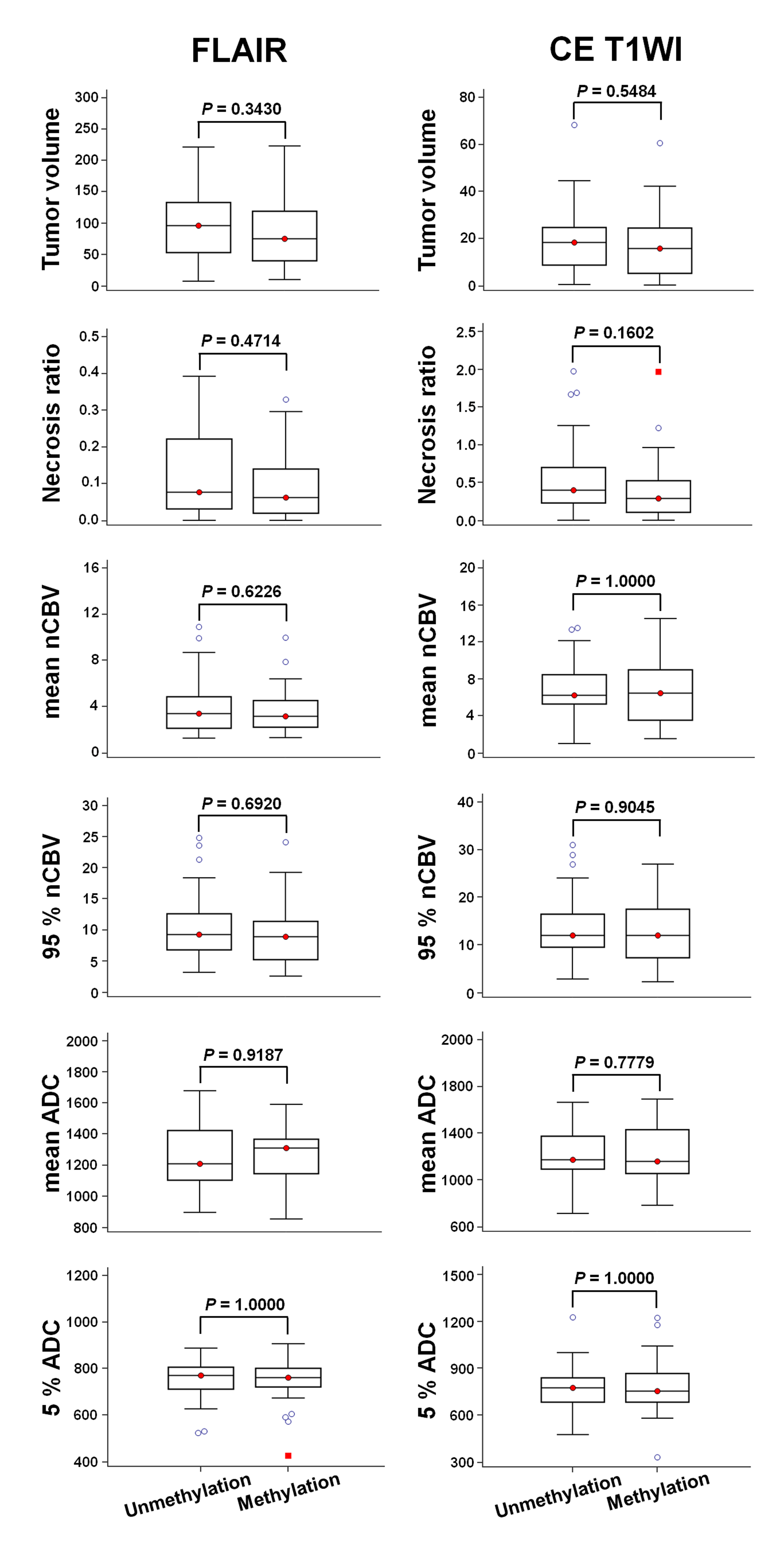
**

**Figure S4.** Comparison of the tumor volume, necrosis ratio, mean and 95 % nCBV, mean and 5 % ADC from FLAIR imaging and CE T1WI according to the MGMT promoter methylation status.

**Table S1. Correlation analysis between quantitative imaging parameters and BCAT1 expression level in IDH1-wildtype and IDH1-mutant groups**

|  |  | IDH1-wildtype (*n* = 60)^*^ | |  | IDH1-mutant (*n* = 9)^+^ | |
| --- | --- | --- | --- | --- | --- | --- |
|  |  | Correlation coefficient | *P* |  | Correlation coefficient | *P* |
| FLAIR | Tumor volume | -0.3473 | 0.0066 |  | 0.1000 | 0.7980 |
|  | Necrosis ratio | 0.0668 | 0.6122 |  | 0.4520 | 0.2220 |
|  | mean nCBV | 0.1981 | 0.1291 |  | 0.4170 | 0.2649 |
|  | 95% nCBV | 0.1220 | 0.3531 |  | 0.4500 | 0.2242 |
|  | mean ADC | -0.3059 | 0.0175 |  | 0.7500 | 0.0599 |
|  | 5% ADC | -0.1103 | 0.4017 |  | 0.4170 | 0.2646 |
|  |  |  |  |  |  |  |
| CE T1WI | Tumor volume | -0.1912 | 0.1433 |  | 0.4330 | 0.2440 |
|  | Necrosis ratio | -0.1387 | 0.2905 |  | 0.4330 | 0.1684 |
|  | mean nCBV | 0.0776 | 0.5556 |  | 0.3500 | 0.3558 |
|  | 95% nCBV | 0.1281 | 0.3292 |  | 0.5500 | 0.1250 |
|  | mean ADC | -0.1049 | 0.4249 |  | 0.6170 | 0.0769 |
|  | 5% ADC | -0.0383 | 0.7717 |  | -0.5670 | 0.1160 |

^*^Pearson correlation analysis was performed for the correlation between the BCAT1 expression level and quantitative imaging parameters.

^+^Spearman rank correlation test was performed for the correlation between the BCAT1 expression level and quantitative imaging parameters.

**Table S2. Reproducibility analysis** between two observers.

|  |  | Observer 1^*^ | Observer 2^*^ | Intraclass correlation coefficient^+^ |
| --- | --- | --- | --- | --- |
| FLAIR | Tumor volume | 89.65 ± 53.28 | 101.22 ± 62.37 | 0.9935 (0.9895, 0.9960) |
|  | Necrosis ratio | 0.11 ± 0.10 | 0.99 ± 0.09 | 0.9965 (0.9944, 0.9978) |
|  | mean nCBV | 3.77 ± 2.21 | 3.99 ± 2.19 | 0.9918 (0.9868, 0.9949) |
|  | 95% nCBV | 10.04 ± 5.17 | 10.06 ± 5.11 | 0.9962 (0.9939, 0.9977) |
|  | mean ADC | 1258 ± 184 | 1262 ± 176 | 0.9958 (0.9932, 0.9974) |
|  | 5% ADC | 906 ± 91 | 738 ± 97 | 0.9861 (0.9776, 0.9914) |
| CE T1WI | Tumor volume | 18.40 ± 13.99 | 18.28 ± 14.10 | 0.9988 (0.9980, 0.9992) |
|  | Necrosis ratio | 0.47 ± 0.46 | 0.42 ± 0.41 | 0.9937 (0.9898, 0.9961) |
|  | mean nCBV | 6.74 ± 3.13 | 6.90 ± 3.13 | 0.9977 (0.9963, 0.9986) |
|  | 95% nCBV | 13.33 ± 6.82 | 13.41 ± 6.67 | 0.9865 (0.9782, 0.9916) |
|  | mean ADC | 1219 ± 227 | 1236 ± 228 | 0.9935 (0.9896, 0.9960) |
|  | 5% ADC | 782 ± 157 | 765 ± 162 | 0.9787 (0.9656, 0.9868) |

^*^ mean ± standard deviation.

^+^ numbers in parenthesis are 95 % confidence intervals.
